# Duplicate origin of the middle cerebral artery: a rare variant

**DOI:** 10.1007/s00276-025-03654-4

**Published:** 2025-05-19

**Authors:** George Triantafyllou, Panagiotis Papadopoulos-Manolarakis, Panagiotis Papanagiotou, George Tsakotos, Maria Piagkou

**Affiliations:** 1 Department of Anatomy, School of Medicine, Faculty of Health Sciences, National and Kapodistrian University of Athens, 75 Mikras Asias str, Goudi, Athens, 11527 Greece; 2 Department of Neurosurgery, General Hospital of Nikaia-Piraeus, Athens, Greece; 3 Department of Radiology, School of Medicine, Aretaieion University Hospital, National and Kapodistrian University of Athens, Athens, Greece

**Keywords:** Middle cerebral artery, Duplicate origin, Temporopolar branch, Variation, Anatomy

## Abstract

The cerebral arterial circle exhibits considerable morphological variability. Variations in the middle cerebral artery (MCA) are infrequent occurrences that can be readily identified via imaging techniques. The current imaging report describes a rare variant of the MCA consisting of a duplicate origin, which was incidentally discovered in a 42-year-old female patient through computed tomography angiography. The duplicated origin of the MCA was identified on the left side of the cerebral arterial circle, forming an arterial ring with the temporopolar branch of the MCA originating from one of its limbs. The remainder of the arterial circle demonstrated no variants. It is essential to distinguish variants of the MCA without conflating them. The present variant is accurately characterized as a ‘duplicate origin”, it should not be confused with fenestrations, and has a reported prevalence of 0.1%. Comprehending such arterial variations is vital prior to undertaking endovascular or neurosurgical procedures in the region.

## Introduction

The cerebral arterial circle, called the “circle of Willis”, serves as the arterial supply for the brain. Its morphological variability has recently been the subject of systematic investigation utilizing imaging techniques widely adopted in routine clinical practice. Various modalities are employed, including computed tomography angiography (CTA), magnetic resonance angiography (MRA), and digital subtraction angiography (DSA).

The cerebral arterial circle is established through the anastomosis of the terminal branches of the carotid and vertebrobasilar systems. The anterior circulation comprises the anterior and middle cerebral arteries (ACA and MCA), whereas the posterior cerebral circulation is represented by the posterior cerebral artery (PCA). The brain’s primary arterial supply source is the MCA, the larger terminal branch of the internal carotid artery (ICA). The surgical classification of the MCA is delineated into four segments: M1 - from its origin to its bifurcation, M2 - the course within the lateral fissure, M3 - from the exit of the lateral fissure, and M4 - the cortical trajectory. The MCA gives rise to several central and cortical branches [4].

*Bergman’s Comprehensive Encyclopedia of Human Anatomic Variations* elucidates the variants observed in the M1 segment and its branching pattern while emphasizing that the M2, M3, and M4 segments lack morphological alterations [7]. In this imaging report, we present a rare variant of the MCA identified incidentally during the evaluation of CTA.

## Anatomic variation

An interesting arterial variant was identified during a retrospective CTA investigation of a Greek adult population sample (affiliation 2). Due to the presence of an unusual arterial variant, the brain CTA of a 42-year-old female patient was further investigated. The investigation was conducted and documented using the Horos software (Horos Project). Evidence was obtained on the multiplanar reconstruction of the axial, coronal, and sagittal slices and their three-dimensional volume reconstruction.

**Fig. 1:**
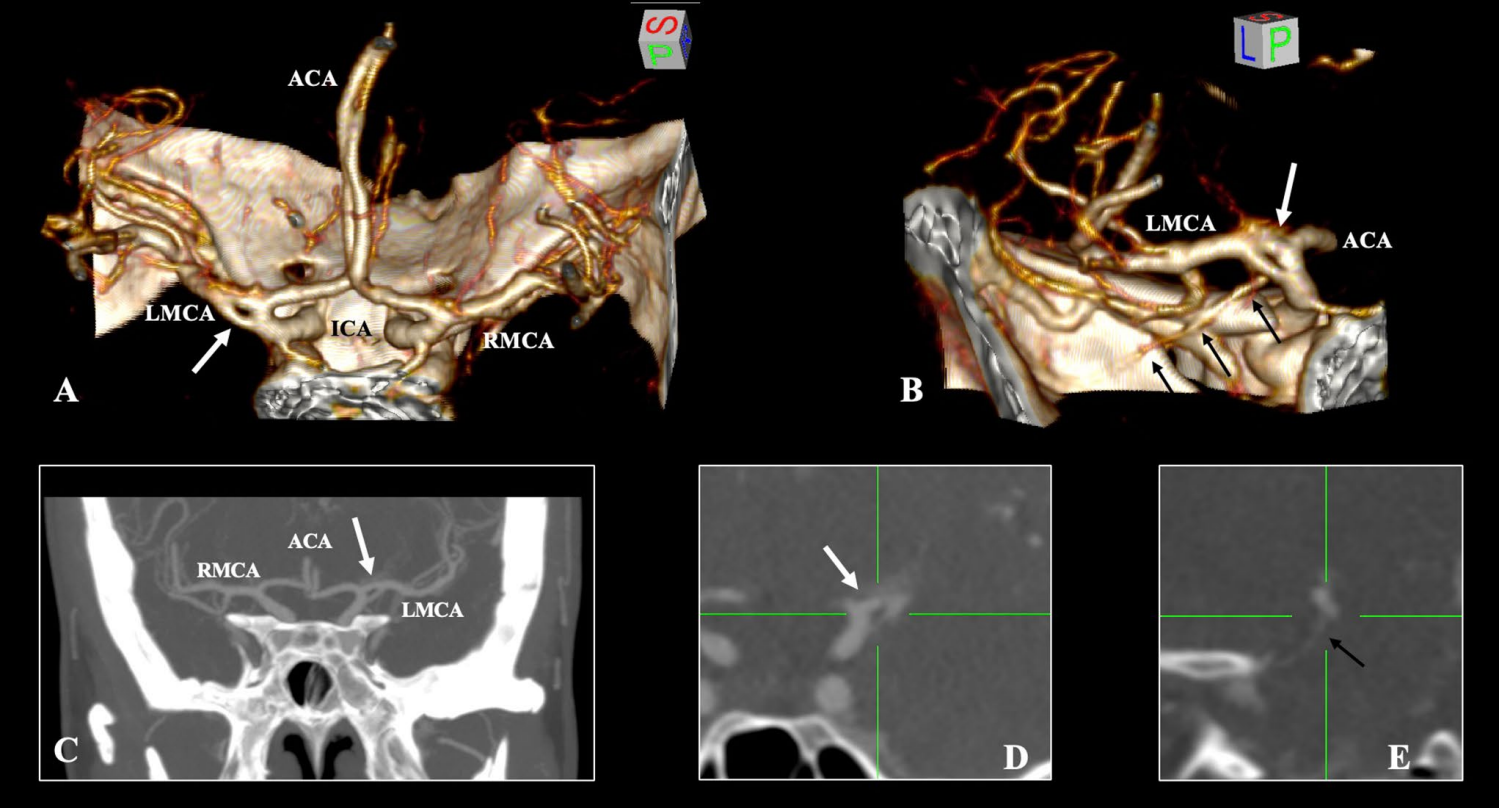
(**A**, **B**) Three-dimensional reconstruction, (**C**, **D**) coronal and (**E**) sagittal slices of the anterior brain circulation. The arterial ring of the left middle cerebral artery (LMCA) is indicated (white arrows in **A**, **B**, **C**, and **D**). The temporopolar branch is marked (black arrows in **B** and **E**). RMCA- right middle cerebral artery, ACA- anterior cerebral artery, and ICA- internal carotid artery

A duplicated origin of the left MCA forming an arterial ring, was detected. The superior limb of the ring exhibited a diameter of 1.47 mm and a length of 3.45 mm. The inferior limb presented a diameter of 1.37 mm and a length of 4.08 mm, giving the temporopolar branch (Fig. 1). Distal to the arterial ring, at a distance of 9.6 mm, the MCA exhibited bifurcation and supplied all subsequent branches.

The MCA did not exhibit any morphological variant on the right side of the cerebral arterial circle. Its diameter measured 2.22 mm at its origin and bifurcated distally after a length of 8.70 mm.

## Discussion

There are limited morphological variants of MCA, including MCA duplication, accessory MCA, duplicate origin, early bifurcation, and fenestration [7, 10]. Sharma et al. [3] conducted an evidence-based systematic review with a meta-analysis of MCA morphological variants. The authors underscored the rarity of these variants, estimating a pooled prevalence of 0.01% for accessory and duplicated MCA and a pooled prevalence of less than 0.01% for fenestrated MCA. It is imperative to underscore that these definitions must be utilized accurately and not conflated.

**Fig. 2:**
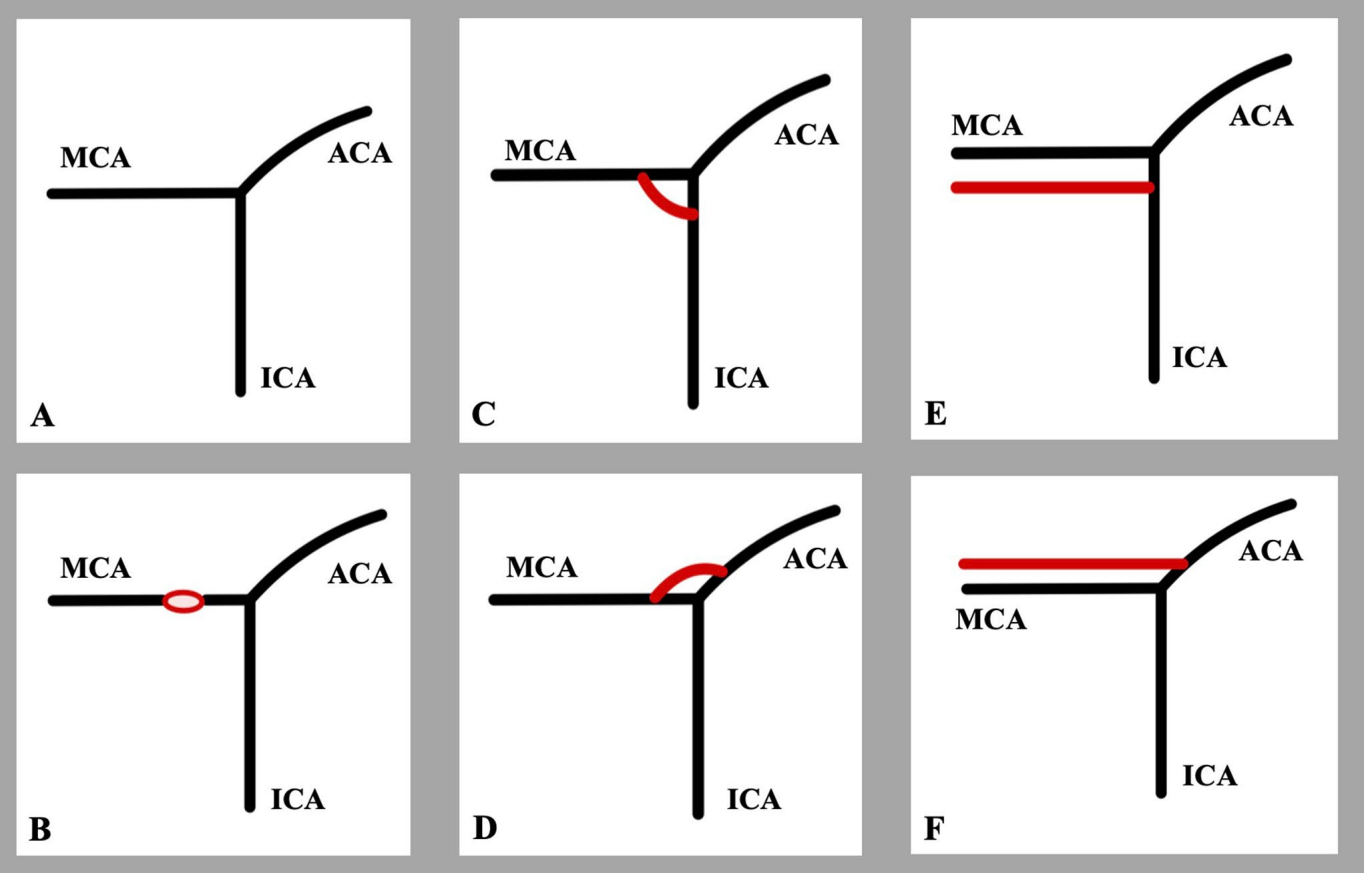
Schematic representation of the middle cerebral artery (MCA) morphological variants according to the literature. **A**- typical (standard) anatomy, **B**- fenestration, **C**- duplicate origin due to regression of duplicated MCA, **D**- duplicate origin due to regression of accessory MCA, **E**- duplicated MCA arising from the ICA, **F**- accessory MCA emanating from the ACA

In the present imaging report, a duplicate origin of the MCA was identified on the left side of a 42-year-old female patient. Although this morphology superficially resembles fenestration, according to the study by Uchino et al., it necessitates clear differentiation [10]. The schematic representation in Fig. 2 illustrates the distinctions between MCA fenestration (Fig. 2B) and MCA duplicate origin (Fig. 2C and D).

The duplicate origin is characterized by two arterial branches originating separately either from the ICA or from the ACA, running parallel to supply the MCA territory, and fusing distally to form an arterial ring [10]. Aneurysms have been known to occur at the MCA duplicate origin. The proximal M1 segment may become occluded due to embolism from the terminal ICA. When the MCA has a duplicate origin, the accessory branch may provide essential collateral circulation when one branch is occluded [10]. Additionally, a temporopolar branch originating from the arterial ring was noted and documented in one of four cases during the study conducted by Uchino et al. [10]. They proposed that the duplicate origin of the MCA might result from the distal fusion of a duplicated MCA (Fig. 2C and E) or an accessory MCA (Fig. 2D and F) [10]. Based on the morphology observed in the current case, it can be hypothesized that it corresponds to a duplicate origin resulting from the fusion of a duplicated MCA (Fig. 2C). Uchino and Kimura [9] identified a frontal branch of the MCA originating from a substantial arterial ring formed by the MCA duplicate origin.

Conversely, fenestration occurs when a single vessel splits and rejoins [8]. In their study, Uchino et al. [10] were the first to delineate the differences between MCA fenestration and duplicate origin, identifying a duplicate origin with a prevalence of 0.1% (4 cases out of 3491 patients), indicative of a scarce variant. In the same sample, a prevalence of 0.2% was reported for fenestration, establishing that the MCA duplicate origin is rarer than the MCA fenestration [10]. MCA fenestrations are related to a higher incidence of aneurysms, particularly at the proximal ends of the fenestrated segments where hemodynamic stress increases [8]. Although this is a rare variation, MCA fenestration could complicate aneurysm clipping procedures to avoid damaging the two channels of the fenestrated segments [2].

Additionally, it is essential to differentiate between the “accessory” and “duplicated” MCA. Teal et al. [6] and Uchino et al. [8] illuminated the differences between these two variations as follows: “duplication” pertains to an anomalous MCA that arises directly from the ICA (Fig. 2E), whereas the “accessory MCA” corresponds to an aberrant branch from the ACA (Fig. 2F) [6, 8]. Uchino et al. [8] recorded the prevalence of “duplicated” and “accessory” MCA at 2.1% and 1.2%, respectively. However, it is crucial to emphasize that “accessory” and “duplicated” MCA are the precise definitions according to the proposal by Kachlík et al. [1] regarding variant terminology. Following this terminology, an “accessory vessel” should be regarded as one that supplies the same territory while originating from the proper vessel. Conversely, an “aberrant vessel” serves the same area but diverges from an alternative source vessel [1]. The embryological origin of the duplication of the MCA is posited to arise from variations in the primitive vascular plexus during fetal development, resulting in incomplete fusion of arterial segments. Notably, Takahashi et al. [5] documented an anastomosis between an “accessory” MCA and a typical MCA. Aneurysms have been identified at the origin of the accessory MCA, whereas duplicated MCA has not been associated with clinical entities [8]. Duplication is often linked to a heightened risk of aneurysms, especially at the bifurcation points, due to the abnormal hemodynamical stress on vessel walls [8]. Variations in vascular anatomy may impact collateral circulation, thereby influencing stroke outcomes. Recognizing this anomaly is crucial during aneurysm clipping or coiling, as well as bypass surgeries. MCA duplication can complicate the diagnosis and treatment of ischemic strokes since the altered vascular anatomy may influence collateral circulation and the effectiveness of endovascular treatments such as thrombectomy [2]. It is essential to recognize the duplication of the MCA during aneurysm clipping, bypass surgeries, or any intracranial procedures involving the Sylvian fissure to prevent inadvertent vascular injuries. MRI and CTA are essential tools for identifying MCA duplication. It is crucial to distinguish it from other vascular anomalies, such as accessory MCA or fenestration, to ensure an accurate diagnosis.

## Conclusions

In the current imaging report, a duplicate origin of the MCA has been identified, forming an arterial ring. A temporopolar branch was noted to arise from the inferior aspect of the aforementioned ring. Four cases in the extant literature exhibit this variant, with a reported prevalence of 0.1%. Notably, only one instance has described the origin of the temporopolar branch. It is imperative to distinguish the duplicate origin of the MCA from MCA fenestration, which can be accurately identified utilizing CTA or MRA.

## Data Availability

No datasets were generated or analysed during the current study.
